# Application of a Novel Au@ZIF-8 Composite in the Detection of Bisphenol A by Surface-Enhanced Raman Spectroscopy

**DOI:** 10.3390/foods12040813

**Published:** 2023-02-14

**Authors:** Yunfei Xie, Xianghui Dong, Nifei Cai, Fangwei Yang, Weirong Yao, Lijun Huang

**Affiliations:** 1 State Key Laboratory of Food Science and Technology, Jiangnan University, No. 1800 Lihu Avenue, Wuxi 214122, China; 2 School of Food Science and Technology, Jiangnan University, No. 1800 Lihu Avenue, Wuxi 214122, China; 3 School of Food and Health, Beijing Technology & Business University, 33 Fucheng Road, Haidian District, Beijing 100048, China; 4 Wuxi Food Safety Inspection and Test Center, 35-210 Changjiang South Road, Wuxi 214142, China

**Keywords:** SERS, metal-organic frameworks, bisphenol A, rapid detection

## Abstract

Bisphenol A (BPA) is an endocrine disruptor which is widely present in fish under the influence of environmental pollution. It is essential to establish a rapid detection method for BPA. Zeolitic imidazolate framework (ZIF-8) is a typical metal-organic framework material (MOFs) with a strong adsorption capacity, which can effectively adsorb harmful substances in food. Combining MOFs and surface-enhanced Raman spectroscopy (SERS) can achieve rapid and accurate screening of toxic substances. In this study, a rapid detection method for BPA was established by preparing a new reinforced substrate Au@ZIF-8. The SERS detection method was optimized by combining SERS technology with ZIF-8. The Raman peak at 1172 cm^−1^ was used as the characteristic quantitative peak, and the lowest detection concentration of BPA was as low as 0.1 mg/L. In the concentration range of 0.1~10 mg/L, the linear relationship between SERS peak intensity and the concentration of BPA was good, and R^2^ was 0.9954. This novel SERS substrate was proven to have great potential in rapidly detecting BPA in food.

## 1. Introduction

Metal-organic frameworks (MOFs) are a kind of material formed by the combination of metal ions and organic ligands, mainly including materials of the Institute Lavoisier (MILs) series [1], zeolitic imidazolate frameworks (ZIFs) series [2], the pocket-channel frameworks (PCNs) series [3], and University of Oslos (UiOs) series [4]. The synthesis methods mainly include the solvothermal method [5], hydrothermal synthesis method [6], microwave synthesis method [7], ultrasonic synthesis method [8], electrochemical synthesis method [9], and the mechanical stirring synthesis method [10]. Compared with other materials (for example, gold/silver nanoparticles and ferric oxide-based materials), MOFs materials have apparent advantages, such as a large specific surface area, a flexible pore diameter, ultra-high porosity, and good chemical stability, so they are widely used in adsorption [11], separation [12], gas storage [13], water purification [14] and catalysis [15]. The ZIF-8 material is a new type of MOF material assembled with Zn^2+^ as the central framework and 2-methylimidazole as the organic ligand. It not only retains the structure like zeolite, but also has the advantages of MOFs material, so it has broad application prospects. Currently, the application of ZIF-8 materials is focused on environmental wastewater treatment, gas adsorption and storage, and drug-sustained release [16]. ZIF-8 can be prepared in high purity by several different synthetic routes, such as conventional solvothermal methods, microwave-assisted methods, acoustic chemistry, mechanochemical stirring, and electrochemical methods [17]. The ideal preparation method for ZIF-8 is to be synthesized in an environmentally friendly manner under easily manipulated conditions [18]. In addition, synthesizing ZIF-8 materials by mechanochemical stirring makes it easier to obtain higher solid product yields than by other methods [19].

Surface-enhanced Raman spectroscopy (SERS) introduced nanotechnology based on ordinary Raman scattering using precious metal nanomaterials as the substrate to enhance the Raman scattering of analytes, thus possessing significant advantages such as high sensitivity and fast screening speed [20]. However, the target molecules often unevenly spread across the substrate, leading to the poor reproducibility of the SERS signals [21]. Moreover, some components contained in the food samples would also adsorb onto the substrate and interfere with the analysis of the target molecules, which limits its further application in the field of food detection [22]. SERS has great application potential in food safety-related detection fields. SERS is widely used to rapidly detect food-borne pathogens, mycotoxins, shellfish toxins, illegal food additives, drug residues, and other toxic and harmful substances in food [23]. Therefore, it is an encouraging attempt to apply ZIF-8 materials together with SERS detection technology in food detection, which can not only increase the application scenes of MOF materials, but also enrich the substrate types of SERS technology [24]. The combined application of MOFs materials and SERS technology is mainly manifested in two aspects. On the one hand, MOFs materials are used in the pretreatment of the SERS detection, and on the other hand, MOFs materials are used in the synthesis of the SERS substrate [25]. The combined application of the two materials has been used in food detection [26].

Bisphenol A (BPA) is an endocrine disruptor. People previously paid more attention to its migration in food packaging. Nowadays, due to environmental pollution, it is also commonly found in fish [27]. How to deal with BPA residues in fresh fish is a problem worth exploring. ZIF-8 is a typical ZIF material and 2-methylimidazole (Hmim) forms a framework material with a square sodalite topology; the structure is highly efficient in the enrichment, determination, and removal of hazardous substances from food [28]. In this study, ZIF-8 material was synthesized by the mechanical stirring method. The advantage of this method is that the whole reaction can be carried out under the condition of solvent-free, avoiding the use of organic solvents, and the reaction time of this method is short, only 10-60 min to obtain a specific yield. The morphology and structure of the material were characterized by a transmission electron microscope (TEM), X-ray diffraction (XRD) and Fourier transform infrared spectroscopy (FT-IR). After the adsorption performance of BPA was verified, ZIF-8 was applied to the pretreatment of the SERS detection to establish a fast detection method of BPA in fish by SERS. The practical application of this method was investigated. At the same time, a new substrate, the Au@ZIF-8 material with a good Raman enhancement effect, was prepared based on the ZIF-8 material.

## 2. Materials and Methods

### 2.1. Schematic Overview of the Experimental Program

**Figure 1:**
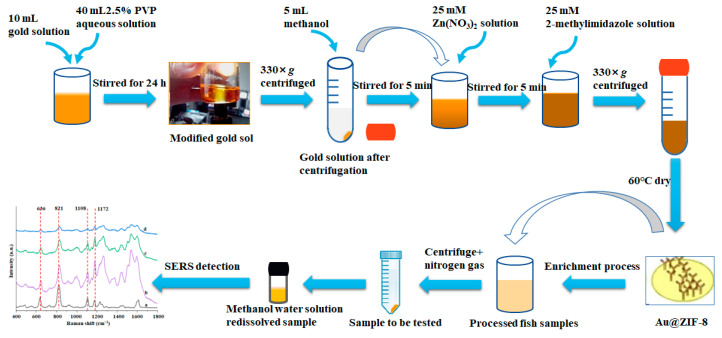
Schematic overview of the experimental program. a. solid BPA standard, b. 3 nm Au@ZIF-8, c. 20 nm Au@ZIF-8, and d. 50 nm Au@ZIF-8.

ZIF-8 can firmly adsorb target molecules, further increasing the contact between target molecules and gold nanoparticles, thus obtaining better detection results. The substrate’s enhancement effect, reproducibility, and stability were investigated using BPA as the target contaminant. A SERS assay for BPA was developed using a new substrate, and the SERS assay conditions were optimized. A standard curve was established, the minimum detection concentration was determined, and then the practical applicability of the method was examined by selecting actual samples (Figure 1).

### 2.2. Chemicals and Reagents

A BPA standard (purity > 98.0%), 2-methylimidazole (AR), and zinc nitrate hexahydrate (AR) were purchased from J&K Scientific Co., LTD (Beijing, China). Sodium hydroxide (AR), trisodium citrate dihydrate (99.0% purity), Na_2_CO_3_ (99.0% purity), anhydrous sodium sulfate (98.0% purity), and Polyvinyl pyrrolidone (Mw = 55,000, 99% purity) were purchased from Shanghai Aladdin Co., LTD (Shanghai, China). Methanol (AR), ethanol (AR), acetonitrile (AR), hydrochloric acid (AR), acetone (AR), ethyl acetate (AR), n-hexane (AR), and HNO₃ (AR) were purchased from Sinopharm Chemical Reagent Co., LTD (Shanghai, China). Potassium tetrachloroaurate (K(AuCl4), purity 97.0%) was purchased from Nanjing Senbeca Biotechnology Co., LTD (Nanjing, China). The above reagents, unless otherwise specified, are Analytical Reagents. The Crucian carp (*Carassius auratus*) fish was purchased from Jingdong (Shanghai, China).

### 2.3. Instruments

A conical flask, a round-bottomed flask and a centrifuge tube were purchased from Shanghai Shendi Glass Co., LTD (Shanghai, China). Further instruments were purchased as follows: ME204E Electronic balance (Mettler Toledo Instruments Co., LTD, Shanghai, China); Shaker oscillator, (Yuezhong Instrument Equipment Co., LTD, Shanghai, China); Milli-Q Ultrapure Water Systems (Millipore, Burlington, MA, USA); GZX-9076MBE Electric thermostatic drying oven (Boxun Industrial Co., LTD, Shanghai, China); 79-1 Heat-Up Magnetic Agitator (Jintan Medical Instrument Factory, Jintan, China); TU-1900 UV-visible spectrophotometer, (Shimadzu Company, Japan); BCD-602W refrigerator (Qingdao Haier Co., LTD, Qingdao, China); PS-10 Endosonic Ultrasonic Cleaner (Jiekang Ultrasonic Equipment Co., LTD, Dongguan, China); BRUCKER D8 ADVANCE X ray apparatus (BRUCKER, Germany); Nicolet IS10 infrared spectrometer (Nicoli Corporation, Salt Lake, UT, USA); SU8020 SEM (Hitachi, Tokyo, Japan); JEM-2100 TEM, (Nippon Electronics Co., Ltd., Chiyoda-ku, Tokyo, Japan); FI-FO785E60X-W Portable Fiber optic Probe Raman spectrometer (Beijing Zhuoli Hanguang Instrument Co., LTD, Beijing, China); ZNCL-GS magnetic heating Oil Bath (Shanghai Yuezhong Instrument Equipment Co., LTD, Shanghai, China); ZGDCY-24 dry nitrogen blowing instrument (Shanghai Zigui Instrument Co., LTD, Shanghai, China); and Waters1525EF High performance liquid chromatography (HPLC) system (Waters, Milford, MA, USA).

### 2.4. Synthesis of Au@ZIF-8

A total of 100 mL of 0.01% K(AuCl_4_) aqueous solution was added to a 250 mL round-bottomed flask that was soaked in aqua regia, and placed in an oil bath at 140 °C. At the same time, it was stirred at 200× *g*, and the reaction temperature was set constant until the solution was boiled. After boiling, 0.75 mL of 1% C_6_H_5_Na_3_O_7_ aqueous solution was added through a pipette. It was observed that the color of the solution changed from transparent to dark and then to brown red in a few minutes. The solution was heated for another 30 min and kept boiling to stabilize the particles in the system. After the reaction, the solution was cooled to room temperature naturally. In this way, the preparation for the gold sol was finished.

A total of 10 mL of prepared gold sol and 40 mL of 2.5% PVP aqueous solution were mixed and stirred for 24 h to obtain PVP-modified gold sol. Fifteen milliliters of gold sol solution that was modified was centrifuged (Beckman Coulter, Inc., California, CA, USA) for 10 min at 330× *g*, and then the supernatant was discarded and 5 mL methanol was added to evenly mix the lower layer precipitation. The above three solutions were selected and added with different volumes of 25 mM Zn(NO_3_)_2_ solution (0.1, 0.8, 4.0 mL) and stirred for 5 min. Then different volumes of 25 mM 2-methylimidazole solution (0.3, 2.4, 12.0 mL) were added, stirred for 5 min, and allowed to stand for 15 min. At the end of the reaction, the solution was centrifuged at 330× *g* for 10 min. After washing with methanol three times, the products were placed in an oven at 60 °C overnight to dry to obtain the Au@ZIF-8 materials with different shell thicknesses. To use, Au@ZIF-8 was dispersed in 5 mL methanol.

Methanol was used to prepare 50 mM Zn(NO_3_)_2_. A total of 50 mL of the above solution and 100 mM of 2-methylimidazole solution were fully and evenly mixed and stirred at room temperature for 2 h. At the end of the reaction, a white suspension was obtained. The above solution was centrifuged at 330× *g* for 10 min and then washed with methanol for three times. The product was dried overnight in an oven at 60℃, and ZIF-8 was obtained after taking out and grinding.

### 2.5. Study on the Optimum Synthesis Conditions of Au@ZIF-8

The reinforced substrates with different shell thicknesses were prepared with 5 µL BPA at a concentration of 50 mg/L and 5 µL Au@ZIF-8 suspension, and the SERS enhanced effect was evaluated by Raman detection. Twelve points were scanned consecutively for reproducibility assessment. In order to evaluate the stability of the synthetic materials, 5 µL BPA solution (50 mg/L) and 5 µL Au@ZIF-8 suspension were mixed, which was detected by Raman spectroscopy (FI-FO785E60X-W portable optical fiber probe Raman spectrometer, Beijing Zolix Instrument Co., Ltd, Beijing, China).

### 2.6. Application of Au@ZIF-8 in SERS Detection

#### 2.6.1. SERS Detection

The optimal detection conditions were as follows: the mixing time was set to 180 s, and the volume ratio of the tested solution to the Au@ZIF-8 suspension was set to 1:3. The optimum solvent ratio was used to prepare the BPA standard solutions of 0.05, 0.1, 0.5, 1, 5, and 10 mg/L, and then SERS detection and analysis were carried out to obtain the standard curve, and the detection limit of this method was determined.

#### 2.6.2. Spiking Recovery Tests

Meanwhile, the content of BPA in fish was detected by spiking recovery tests. When the ZIF-8 material was used as the substrate, the spiked concentration of BPA in fish was 0.5, 1.0 and 5.0 mg/kg; when Au@ZIF-8 material was used as the substrate, the spiked concentration of BPA in fish was 0.1, 0.2 and 1.0 mg/kg. Then the content of BPA in the fish samples was determined by the SERS method established in this study. The parallel experiment was repeated three times, and the recovery rate was calculated.

#### 2.6.3. Detection of BPA in Fish Samples

Ordinary treatment: a total of 5.00 (±0.05) g of fresh fish was accurately weighed into the centrifuge tube. Then, 2 mL of the BPA standard solution at different concentrations, 3 mL of Na_2_CO_3_ solution (10.0% mass fraction) and 2.0 g of anhydrous Na_2_SO_4_ were added and fully mixed by vortex for 1 min. Ten milliliters of extraction solvent were also added, mixed again by vortex combined with ultrasonic, and finally centrifuged at 330× *g* for 10 min. The upper layer of the organic solvent was transferred to the centrifuge tube, and 10 mL of extraction solvent was used for repeated extraction. The two extracts were combined, concentrated to near dry by nitrogen gas, and redissolved by a methanol solution. The redissolved solution was used for SERS detection.

Enrichment of Au@ZIF-8 on BPA: a total of (5.00 ± 0.05) g of fresh fish was accurately weighed into the centrifuge tube, and then 2 mL of the BPA standard solution with different concentrations was added into the centrifuge tube. Then, 3 mL of the Na_2_CO_3_ solution with 10.0% mass fraction and 2.0 g of anhydrous Na_2_SO_4_ were also added in turn, and fully mixed by vortex for 1 min, and then 10 mL of the extraction solvent was also added, mixed again by vortex combined with ultrasonic, and centrifuged at 330× *g* for 10 min. The upper layer of the organic solvent was transferred to the centrifuge tube, and the samples were extracted with 10 mL of the extraction solvent. The two extracts were combined, concentrated to near dry by nitrogen blowing, then ultrapure water was used for resolution. A total of 20 mg of the Au@ZIF-8 material was added to the redissolved solution and shaken in a shaker at room temperature for 30 min, and eluted after centrifugation. The eluent was then dried by nitrogen blowing, then the methanol solution was used to redissolve it. The redissolved solution was used to enrich BPA in the fish samples with Au@ZIF-8, and the recovery experiment was carried out.

### 2.7. Comparison of Au@ZIF-8 SERS Detection with HPLC

High-Performance Liquid Chromatography (HPLC) was used to evaluate and compare the detection results of the SERS method. Based on previous studies [29], HPLC detection conditions were established. The column of the mobile phase of Waters SunFire^®^C18 (4.6 × 250 mm, five μm) was determined as methanol/water (3:1, *v*/*v*), the flow rate was 1 mL/min, the column temperature was 40 °C, the injection volume was ten μL, and the UV detector was 280 nm.

After establishing the HPLC detection method for BPA, the standard solutions with concentrations of 0.1, 0.5, 1.0, 5.0, and 10 mg/L were used to establish the BPA detection curve. In the range of 0.1–10 mg/L, the concentration of BPA was used as the abscissa, and the corresponding chromatographic peak integral area was used as the ordinate to establish the standard curve, which was used for the subsequent detection in actual samples, and to evaluate the accuracy of SERS detection results.

### 2.8. Data Processing

The charts were drawn mainly by Origin 2018 (Originlab Corporation, Northampton, NC, USA) drawing software, and the data were processed by SPSS 26.0 (International Business Machines Corporation, Armonk, NY, USA). Where applicable, the degree of variance was explained by the relative standard deviation (RSD), which refers to the ratio of standard deviation to the arithmetic mean of measurement results and is usually used to express the precision of analytical test results.

## 3. Results and Discussion

### 3.1. Characterization of Au@ZIF-8

**Figure 2:**
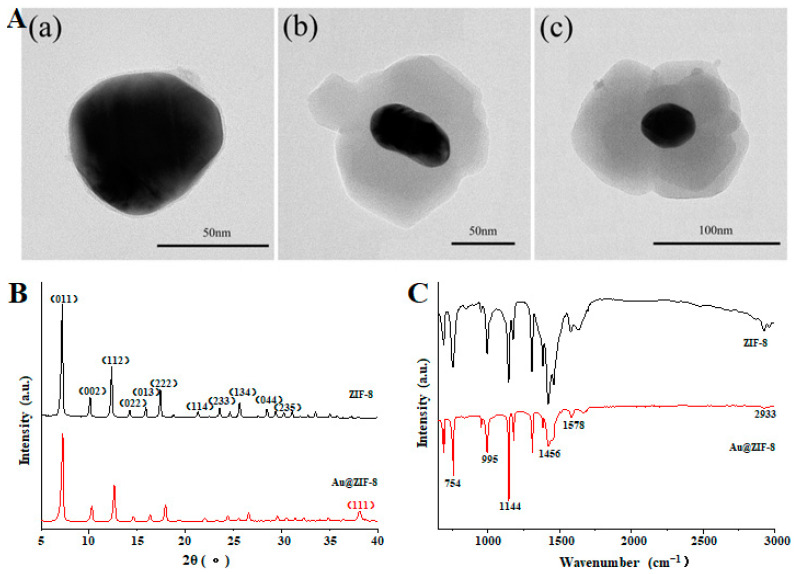
(**A**) TEM of synthesized ZIF-8 and Au@ZIF-8 ((**a**) 3 nm, (**b**) 20 nm, and (**c**) 50 nm Au@ZIF-8); (**B**) XRD and (**C**) FT-IR pattern of synthesized ZIF-8 and Au@ZIF-8.

The synthesized ZIF-8 and Au@ZIF-8 were characterized by TEM. The results are shown in Figure 2A where the black core represents Au particles and the gray shell ZIF-8 material [30]. Au@ZIF-8 materials with shell thicknesses of 3, 20, and 50 nm were synthesized by adjusting the additional amount of zinc nitrate and 2-methylimidazole. The crystal structure of ZIF-8 was polyhedral, and the particle size was relatively uniform (the diameter was about 50 nm). The gray ZIF-8 shell wrapped around the black Au core and had a complete core-shell structure. Because the shell of ZIF-8 is relatively thin, it is also believed to further promote the formation of SERS hot spots between gold nanoparticles [31].

In Figure 2B, XRD shows that the synthesized ZIF-8 has several diffraction peaks, among which the sharpest peak is at 2 θ = 7.5°, indicating that the synthesized material has high crystallinity. Compared to ZIF-8, a new diffraction peak appears for Au@ZIF-8 at 38.1°, which is the diffraction peak of Au, corresponding to the plane (111) [32].

According to the FT-IR results in Figure 2C, the absorption peak at 421 cm^−1^ is caused by the stretching vibration of Zn-N; the characteristic absorption peaks at 995 cm^−1^ and 1144 cm^−1^ are attributed to the vibration of C-N bond, and the absorption peak at 1578 cm^−1^ is caused by the vibration of C=N bond in imidazole ring [33,34]. In addition, it can also be found that there is a broad infrared absorption peak at 3442 cm^−1^, which is the characteristic absorption peak of the O-H bond in water molecules, indicating that there may also be some free water in the ZIF-8 material [35]. The FT-IR results of Au@ZIF-8 showed that the infrared spectrum of the synthesized material is like that of ZIF-8, and there is no impurity peak formation, indicating that the synthesized material has a complete structure and no impurity interference [36].

### 3.2. The Optimum Synthesis Conditions Properties of Au@ZIF-8

**Figure 3:**
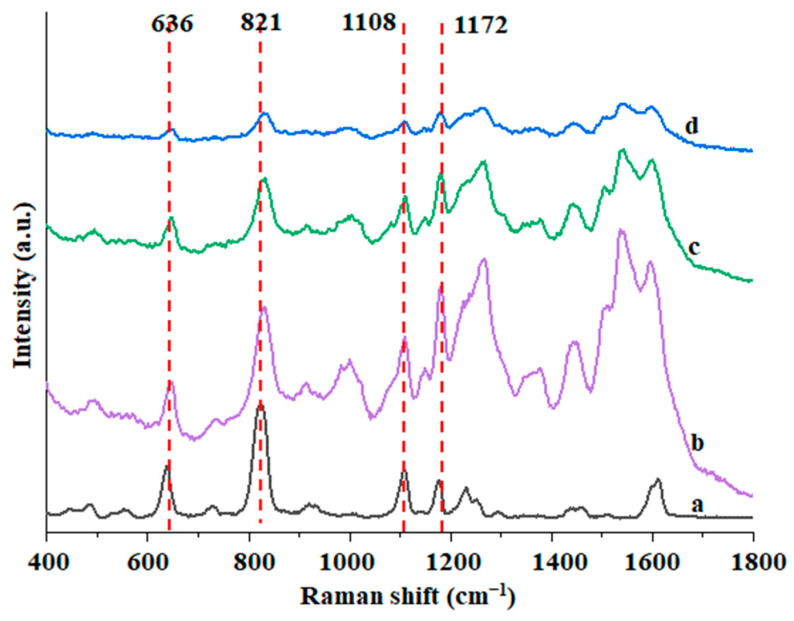
SERS spectra of BPA in Au@ZIF-8 nanoparticles in methanol solution (a. solid BPA standard, b. 3 nmAu@ZIF-8, c. 20 nm Au@ZIF-8, and d. 50 nm Au@ZIF-8).

Layer thickness. Figure 3 shows the influence of Au@ZIF-8 with different shell thicknesses on the SERS enhanced effect. The Raman enhancement effect is enhanced with the decrease of the ZIF-8 layer thickness because the target molecules adsorbed by the material can directly make complete contact with the gold nanoparticles, which dramatically enhances the Raman signal of the target molecules [32]. Therefore, Au@ZIF-8 with an outer layer of 3 nm was selected as the detection thickness.

SERS reproducibility. When measuring the stability of Au@ZIF-8, it can be seen from Figure S1 that the position of the characteristic peak and Raman intensity of BPA did not change much within 30 days and remained in a stable state with the average RSD of SERS intensity of five characteristic peaks at 6.01%, which shows that Au@ZIF-8 has good reproducibility [37].

SERS storage stability. After different storage days, the prepared Au@ZIF-8 was fully mixed with the solution of BPA to be detected, and the SERS spectrum of BPA was collected. The results are shown in Figure S1. It can be seen from Figure S1 that in the first 30 days, the characteristic peak and intensity of BPA did not change much, and remained in a stable state, which indicates that the Au@ZIF-8 long-lasting SERS enhancement effect can be maintained at the initial stage [37]. When the time exceeds 30 days, the characteristic peak of BPA can still be clearly identified, but the corresponding SERS intensity declines. When the time reaches 60 days, its SERS intensity is only about one-third of the initial. The results show that Au@ZIF-8 within 30 days after synthesis, the SERS stability is excellent.

### 3.3. The Optimum SERS Detection Conditions of Au@ZIF-8

**Figure 4:**
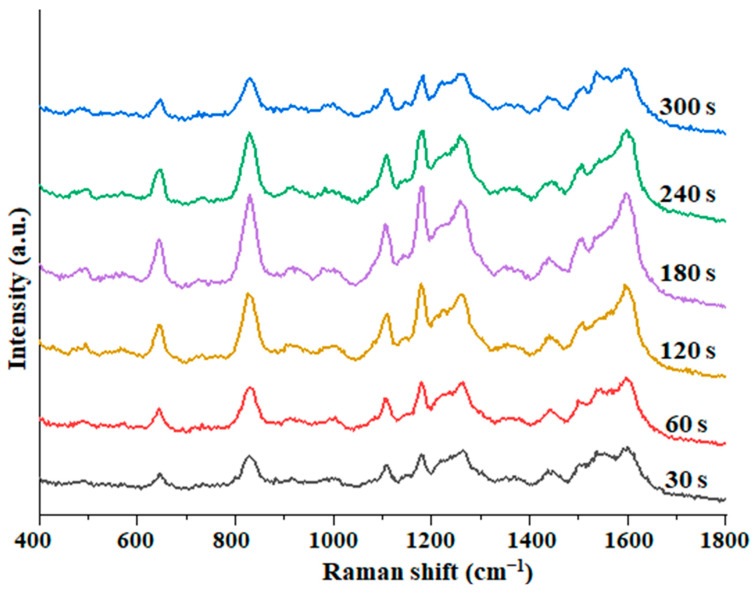
SERS spectra of BPA in methanol solution at different adsorption times.

Mixing time. As can be seen from Figure 4, the SERS intensity of BPA increased with the mixing time from 30 to 180 s. The SERS intensity continued to decrease when the time exceeded 180 s. On the other hand, it could be that at the beginning, as time goes on, the BPA molecules in the solution continue to be adsorbed. At the same time, as the solvent volatilizes, more and more BPA molecules come into contact with the substrate, and the number of BPA molecules adsorbed per unit substrate increases, and the SERS signal also becomes stronger [32]. When the solvent evaporates to a certain degree, it will form “dry patches”, the system is in a state of excessive accumulation, and the SERS intensity is also weakened [24]. So, the mixing detection time was selected as 180 s.

**Figure 5:**
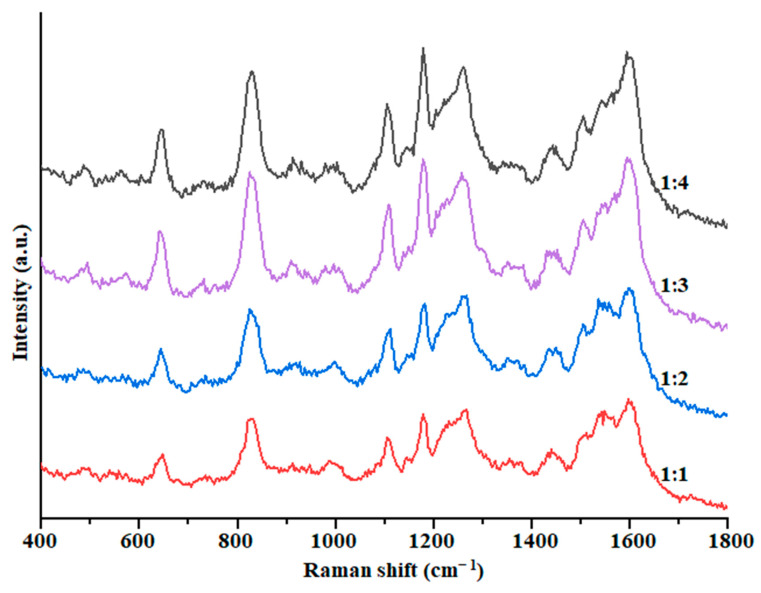
Effect of the solution—substrate ratio on the SERS spectrum of BPA in methanol solution.

Different ratio of solution. The ratio of solution and substrate can also influence the SERS effect [38], which was optimized; the results are shown in Figure 5. As can be seen, when the solution was mixed with the Au@ZIF-8 suspension at a ratio of 1:3, the SERS enhanced effect was the best and relatively ideal and has a smaller amount of substrate used compared with 1:4. Therefore, the mixing ratio was fixed as 1:3 in the subsequent experiments.

### 3.4. SERS Detection of Au@ZIF-8 for BPA

**Figure 6:**
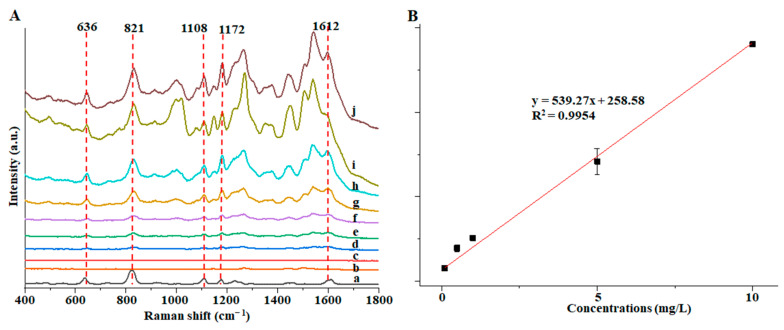
(**A**) SERS spectra of BPA in the solid state (a), in methanol (b), and in 0.05 (c), 0.1 (d), 1 (e), 5 (f), 10 (g), 25 (h), 50 (i) and 100 (j) mg/L Au@ZIF-8 solution; (**B**) Intensity of the BPA peak at 1172 cm^−1^ as a function of BPA concentration (0.05, 0.1, 1, 5 and 10 mg/L).

SERS detection was carried out on different concentrations of BPA by the optimal detection method of Au@ZIF-8. Figure 6 showed the Raman peaks at 636, 821, 1108, 1172, and 1612 cm^−1^ that were used to determine the minimum detection limit, because these Raman peaks were greatly enhanced. Among them, 636 cm^−1^ is a skeleton vibration of para-substituted benzene, 821 cm^−1^ is a vibration of para-substituted benzene, 1108 cm^−1^ is a C=C stretching vibration, and 1172 cm^−1^ is a vibration of para-substituted benzene [39]. As can be seen in Figure 6A, with the gradual decrease in the concentration of BPA solution, the SERS signal intensity of the five characteristic Raman peaks all decreased. When the concentration was 0.1 mg/L, the five Raman peaks could be clearly identified. When the concentration continued to decrease to 0.05 mg/L, the five Raman peaks could not be identified (the curve represented by c in Figure 6A). The possible reason may be that the concentration of BPA is already deficient. The Raman spectrum information of a tiny amount of BPA cannot be captured and enhanced by the Au@ZIF-8 material [31]. Therefore, the detection limit of this method is 0.1 mg/L. When the concentration of BPA was in the range of 0.1~10 mg/L, there was a good linear relationship between the peak intensity at 1172 cm^−1^ and the solution concentration, and the R^2^ could reach 0.9954 (Figure 6B).

The different SERS effects between ZIF-8 and Au@ZIF-8 are listed in Table 1. It can be seen that the SERS substrate prepared by Au@ZIF-8 was better than ZIF-8, including a lower detection limit, more comprehensive linear range, and more significant R^2^.

### 3.5. Detection of Actual Samples

**Figure 7:**
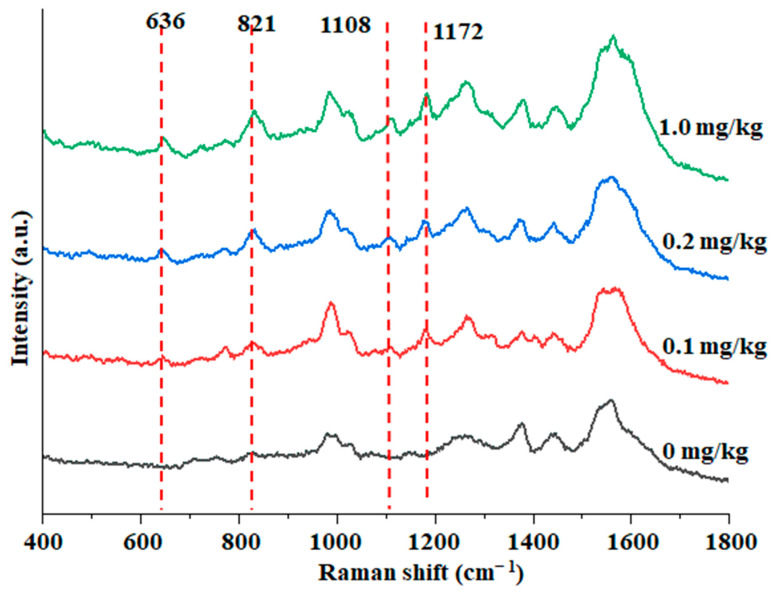
SERS spectra of the fish meat samples with different spiked BPA concentrations.

Detection of BPA in fish by SERS. Due to the severe and extensive pollution range of BPA, the residue of BPA in aquatic products, especially fish, is inevitable [27]. The enrichment effect of Au@ZIF-8 in the pretreatment of fish was investigated by using BPA samples with different spiked concentrations in Figure 7. When Au@ZIF-8 was used as the reinforced substrate, the spiked fish sample well identified according to the Raman characteristic peak of BPA at 1172 cm^−1^, indicating that the Au@ZIF-8 SERS detection method could detect the actual sample contaminated with BPA at 0.1 mg/kg.

The enrichment effect of ZIF-8 and Au@ZIF-8 in fish pretreatment was investigated using BPA samples with different spiked concentrations. The results are shown in Table 2. It is found that the recovery rate, accuracy, and precision (RSD value) meet the requirements.

**Figure 8:**
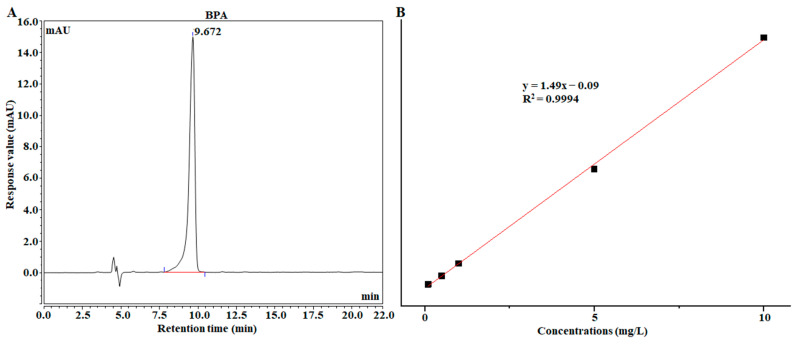
(**A**) HPLC detection results of BPA; (**B**) Standard curve between peak area and concentration in the range of 0.1–10 mg / L.

Detection of BPA by HPLC. Many other detection techniques, such as gas chromatography-tandem mass spectrometry (GC-MS), liquid chromatography (LC) and liquid chromatography-mass spectrometry (LC-MS), electrochemical analysis, and enzyme-linked immunosorbent assays can be used for the detection of BPA [40,41]. The SERS method and HPLC method were used to detect fish samples with different spiked concentrations of BPA, and the results of the HPLC method are shown in Figure 8. Moreover, the accuracy of the SERS method was investigated, and the results are shown in Table 3.

Three supplemental levels of 0.1, 0.2, and 1 mg/kg were selected for the spiked experiment. The results show that the average spiked recovery of BPA in actual samples was 99.68–102.35%, the RSD value was 3.28–7.58%, and the deviation was −1.68–3.43%, indicating that the detection method based on this substrate has high accuracy. The detection results of HPLC were consistent with those of SERS. Compared with the HPLC method, the SERS method established in this paper had the advantage of fast detection, so this method could be used to rapidly detect BPA residues in fish. Once again, the SERS method can be used to quickly, economically, and simply determine the chemical hazards in food [23].

### 3.6. Comparison of Au@ZIF-8 SERS Detection with Other SERS Methods

The method established in this study was compared with other SERS detection methods for BPA, and the results are shown in Table 4, and our new method can be applied to the detection of BPA in real food samples with a low detection limit.

As can be seen from Table 4, compared with other SERS detection methods, the detection limit of the method established with Au@ZIF-8 as the enhanced substrate was relatively lower. Meanwhile, it could be found that some SERS methods selected simple food substrates such as drinking water as actual samples [46,47], and lacked research on complex food substrates such as fish. The detection limit of this method meets the EU regulation 2004/19/EC limit for BPA residues in food (0.6 mg/kg). At present, China has not regulated the residue of BPA in fish meat. So, this method can not only be used as a quick detection method practical application, but also provide a supplementary reference for the establishment of the related detection standard. Subsequent research can achieve a more sensitive and faster detection of food pollutants through applying the Au@ZIF-8 substrate combined with chemometrics methods, such as machine learning and artificial intelligence, which will also have better significance for food safety and human health protection [48]. In addition, we can also consider further improving the characteristics and performance of Au@ZIF-8 nanoparticles in the future, which can be used as a diagnostic tool and also be helpful for the determination of physiological index molecules and disease biomarkers [49]. In a word, the Au@ZIF-8 material has a wide range of application potential under the background of today’s “One Health”, which deserves further detailed exploration.

## 4. Conclusions

The Au@ZIF-8 Raman reinforced substrate with shell layer within 3~50 nm was further synthesized, and the detection limit of the BPA solution was up to 0.1 mg/L. In the detection of actual fish, the average recoveries were 99.68–102.35%, and the RSD values were 3.28–7.58%, which were consistent with the results of HPLC. There is no limited standard of BPA in fish, so this novel and rapid method can provide a supplementary reference for establishing a related standard. Moreover, with the help of new substrate materials such as Au@ZIF-8, the application of the SERS rapid detection method in food safety and human health can also be considered to be full of bright potential.

In this study, the combination of MOFs materials and SERS technology to detect food pollutants has achieved specific results, but there are still shortcomings, such as:

1. There may be many pollutants in food, and the combined method of MOFs and SERS can be established for more contaminants in the future.
2. The adsorption mechanism of ZIF-8 and the enhancement mechanism of Au@ZIF-8 have only been preliminarily discussed, and the related mechanism can be further explored in the future.

## Figures and Tables

**Table 1:** Comparison with the SERS effect between ZIF-8 and Au@ZIF-8.

	Detection Limit (mg/L)	Linear Range (mg/L)	R^2^
ZIF-8	1.0	1–50	0.9846
Au@ZIF-8	0.1	0.1–10	0.9954

**Table 2:** The recovery and RSD of BPA.

Material	Spike Concentration (mg/kg)	Average Recovery Rate (%)	RSD ^a^ Value (%)
ZIF-8	0.5	80.35	2.43
	1.0	85.57	9.42
	5.0	84.47	11.81
Au@ZIF-8	0.1	102.35	7.58
	0.2	99.68	6.14
	1.0	100.82	3.28

^a^ Note: RSD, relative standard deviation.

**Table 3:** The recovery rate of BPA spiked with different addition levels and methods.

Addition Level (mg/kg)	SERS Recovery Rate (%)	HPLC Recovery Rate (%)	Deviation from HPLC Results (%)
0.100	102.4	99.0	3.43
0.200	99.7	101.4	−1.68
1.000	100.8	100.3	0.50

**Table 4:** Comparison of different SERS detection methods of BPA.

Reinforced Substrate	Actual Sample	Detection Limit	Reference
Au@ZIF-8	Fish	0.10 mg/L	This study
AuNPs	PC packaging	7.50 mg/kg	[42]
AgNPs	/	1.00 mg/L	[43]
Au-Ag film	/	0.50 mg/L	[44]
MIPs@AgNPs	Polycarbonate toys	0.01 mg/L	[45]
Functionalized Au-Ag nanoparticles	Soda water	0.05 mg/L	[46]
MIP-ir-AuNPs	Drinking water	0.12 mg/L	[47]

## Data Availability

All related data and methods are presented in this paper. Additional inquiries should be addressed to the corresponding author.

## Supplementary Materials

The following supporting information can be downloaded at: https://www.mdpi.com/article/10.3390/foods12040813/s1, Figure S1: Effect of Au@ZIF-8 on enhancing the Raman spectrum signal of BPA with its storage time.

## References

[1] Khan S., Guan Q., Liu Q., Qin Z., Rasheed B., Liang X., Yang X. (2022). Synthesis, modifications and applications of MILs Metal-organic frameworks for environmental remediation: The cutting-edge review. Sci. Total. Environ..

[2] Kamali K., Joseph B., Narayana C. (2022). Stability of zeolitic imidazolate frameworks (ZIF-7) under high pressures and its implications on storage applications of ZIFs. J. Solid State Chem..

[3] Ma S., Zhou H.-C. (2006). A metal-organic framework with entatic metal centers exhibiting high gas adsorption affinity. J. Am. Chem. Soc..

[4] Ru J., Wang X., Wang F., Cui X., Du X., Lu X. (2021). UiO series of metal-organic frameworks composites as advanced sorbents for the removal of heavy metal ions: Synthesis, applications and adsorption mechanism. Ecotoxicol. Environ. Saf..

[5] Jiang Y., Dong J., Li R., Sun F., Wu H. (2022). Channel regulation through solvents for Cd-MOFs based on p-methoxyphenyl imidazole dicarboxylate: Synthesis, crystal structure, fluorescence, and explosive identification. J. Chin. Chem. Soc..

[6] Ueda S., Hirai Y., Sun H., Matsushima Y., Masuhara A., Shiroishi H., Yoshida T. (2018). Hydrothermal Synthesis and Electrochemical Evaluation of Zn-Tmla MOFs Employing Trimellitic Acid. Electrochemical Society Meeting Abstracts 4dms18.

[7] Elsabawy K.M., Fallatah A.M. (2018). Microwave assisted synthesis and molecular structure visualization of ultrahigh surface area Ni-6,6′-dibromo-indigo coordinated polymeric MOFs stabilized via hydrogen bonding. Inorg. Chem. Commun..

[8] Saidi M., Benomara A., Mokhtari M., Boukli-Hacene L. (2020). Sonochemical synthesis of Zr-fumaric based metal-organic framework (MOF) and its performance evaluation in methyl violet 2B decolorization by photocatalysis. React. Kinet. Mech. Catal. Lett..

[9] Gangu K.K., Maddila S., Mukkamala S.B., Jonnalagadda S.B. (2016). A review on contemporary Metal-Organic Framework materials. Inorg. Chim. Acta.

[10] Braga D., Curzi M., Johansson A., Polito M., Rubini K., Grepioni F. (2006). Simple and quantitative mechanochemical preparation of a porous crystalline material based on a 1D coordination network for uptake of small molecules. Angew. Chem. Int. Ed..

[11] Huang Z., Xiong C., Ying L., Wang W., Wang S., Ding J., Lu J. (2022). A post-functional Ti-based MOFs composite for selective removal of Pb (II) from water. J. Hazard. Mater..

[12] Deng H., Chang Z., Qiu F., Qiao Y., Yang H., He P., Zhou H. (2020). A Safe Organic Oxygen Battery Built with Li-Based Liquid Anode and MOFs Separator. Adv. Energy Mater..

[13] Colorado-Peralta R., Rivera-Villanueva J.M., Mora-Hernández J.M., Morales-Morales D., Alfonso-Herrera L. (2022). An overview of the role of supramolecular interactions in gas storage using MOFs. Polyhedron.

[14] Hui Y.F. (2019). Study on synthesis of a novel MOF-Cd material and adsorption performance of methylene blue. Mod. Salt Chem. Ind..

[15] Jiang H., Wang Q., Wang H., Chen Y., Zhang M. (2016). MOF-74 as an Efficient Catalyst for the Low-Temperature Selective Catalytic Reduction of NO*_x_* with NH_3_. ACS Appl. Mater. Interfaces.

[16] Trickett C.A., Helal A., Al-Maythalony B.A., Yamani Z.H., Cordova K.E., Yaghi O.M. (2017). The chemistry of metal–organic frameworks for CO2 capture, regeneration and conversion. Nat. Rev. Mater..

[17] Lee Y.-R., Jang M.-S., Cho H.-Y., Kwon H.-J., Kim S., Ahn W.-S. (2015). ZIF-8: A comparison of synthesis methods. Chem. Eng. J..

[18] Wang Q., Sun Y., Li S., Zhang P., Yao Q. (2020). Synthesis and modification of ZIF-8 and its application in drug delivery and tumor therapy. RSC Adv..

[19] Kukkar P., Kim K.-H., Kukkar D., Singh P. (2021). Recent advances in the synthesis techniques for zeolitic imidazolate frameworks and their sensing applications. Coord. Chem. Rev..

[20] Guo Z., Wang M., Barimah A.O., Chen Q., Li H., Shi J., El-Seedi H.R., Zou X. (2021). Label-free surface enhanced Raman scattering spectroscopy for discrimination and detection of dominant apple spoilage fungus. Int. J. Food Microbiol..

[21] Duan N., Qi S., Guo Y.C., Xu W., Wu S., Wang Z. (2020). Fe_3_O_4_@Au@Ag nanoparticles as surface-enhanced Raman spectroscopy substrates for sensitive detection of clenbuterol hydrochloride in pork with the use of aptamer binding. LWT.

[22] Zhang Y., Zhao S., Zheng J., He L. (2017). Surface-enhanced Raman spectroscopy (SERS) combined techniques for high-performance detection and characterization. TrAC-Trends Anal. Chem..

[23] Yang F., Wang C., Yu H., Guo Y., Cheng Y., Yao W., Xie Y. (2022). Establishment of the thin-layer chromatography-surface-enhanced Raman spectroscopy and chemometrics method for simultaneous identification of eleven illegal drugs in anti-rheumatic health food. Food Biosci..

[24] Kim H., Trinh B.T., Kim K.H., Moon J., Kang H., Jo K., Akter R., Jeong J., Lim E.-K., Jung J. (2021). Au@ZIF-8 SERS paper for food spoilage detection. Biosens. Bioelectron..

[25] Xue X., Chen L., Wang C., Zhao C., Wang H., Ma N., Li J., Qiao Y., Chang L., Zhao B. (2021). Highly sensitive SERS behavior and wavelength-dependence charge transfer effect on the PS/Ag/ZIF-8 substrate. Spectrochim. Acta A.

[26] Yang J., Pan M., Yang X., Liu K., Song Y., Wang S. (2022). Effective adsorption and in-situ SERS detection of multi-target pesticides on fruits and vegetables using bead-string like Ag NWs@ZIF-8 core-shell nanochains. Food Chem..

[27] Schiano M.E., Abduvakhidov A., Varra M., Albrizio S. (2020). Aptamer-Based Biosensors for the Analytical Determination of Bisphenol A in Foodstuffs. Appl. Sci..

[28] Yang L., Nie L.-Q., Wang J., Li C.-Y., Liu J.-M., Wang S. (2022). ZIF-8 sacrificial-templated hollow COF architectures enabled highly efficient enrichment, determination and regulation of food hazards from infant formulas. Food Chem..

[29] Aristiawan Y., Aryana N., Putri D., Styarini D. (2015). Analytical Method Development for Bisphenol a in Tuna by Using High Performance Liquid Chromatography-UV. Procedia Chem..

[30] Wang B.-X., Duan G., Xu W., Xu C., Jiang J., Yang Z., Wu Y., Pi F. (2022). Flexible surface-enhanced Raman scatting substrates: Recent advances in their principles, design strategies, diversified material selections and applications. Crit. Rev. Food Sci. Nutr..

[31] Li C., Huang Y., Li X., Zhang Y., Chen Q., Ye Z., Alqarni Z., Bell S.E.J., Xu Y. (2021). Towards practical and sustainable SERS: A review of recent developments in the construction of multifunctional enhancing substrates. J. Mater. Chem. C.

[32] Chen Q.-Q., Hou R.-N., Zhu Y.-Z., Wang X.-T., Zhang H., Zhang Y.-J., Zhang L., Tian Z.-Q., Li J.-F. (2021). Au@ZIF-8 core–shell nanoparticles as a SERS substrate for volatile organic compound gas detection. Anal. Chem..

[33] Lin K.-Y.A., Chang H.-A. (2015). Efficient Adsorptive Removal of Humic Acid from Water Using Zeolitic Imidazole Framework-8 (ZIF-8). Water Air Soil Pollut..

[34] Jian M., Liu B., Zhang G., Liu R., Zhang X. (2015). Adsorptive removal of arsenic from aqueous solution by zeolitic imidazolate framework-8 (ZIF-8) nanoparticles. Colloids Surf. Physicochem. Eng. Asp..

[35] Wu Z., Wang X., Qiu J., Liu C., Yu Z., Zhang J., Qiu Z. (2022). In-situ uniform growth of ZIF-8 on 3D flower-like NiCoLDH microspheres to enhance tetracycline and doxycycline removal from wastewater: Anti-interference and stability tests. Sep. Purif. Technol..

[36] Zhang Y., Zhang Z., Rong S., Yu H., Gao H., Ding P., Chang D., Pan H. (2020). Electrochemical immunoassay for the carcinoembryonic antigen based on Au NPs modified zeolitic imidazolate framework and ordered mesoporous carbon. Microchim. Acta.

[37] Huang K., Gong S., Zhang L., Zhang H., Li S., Ye G., Huang F. (2021). Ultrathin ZIF-8 wrapping on Au-dotted Ag-nanowires for highly selective SERS-based CO_2_ gas detection. Chem. Commun..

[38] Xing L., Wang C., Cao Y., Zhang J., Xia H. (2021). Macroscopical monolayer films of ordered arrays of gold nanoparticles as SERS substrates for *in situ* quantitative detection in aqueous solutions. Nanoscale.

[39] Xue J.-Q., Li D.-W., Qu L.-L., Long Y.-T. (2013). Surface-imprinted core–shell Au nanoparticles for selective detection of bisphenol A based on surface-enhanced Raman scattering. Anal. Chim. Acta.

[40] Deng Z.-H., Li N., Jiang H.-L., Lin J.-M., Zhao R.-S. (2019). Pretreatment techniques and analytical methods for phenolic endocrine disrupting chemicals in food and environmental samples. TrAC-Trends Anal. Chem..

[41] Petersen M., Yu Z., Lu X. (2021). Application of Raman spectroscopic methods in food safety: A review. Biosensors.

[42] Yu X.J., Gu Y.Y., Feng R., Ge X.Y., Chen M.Y., Ni M.L., Hu X.L., Zhong Y.Y. (2014). Simultaneously determining alkylphenols and bisphenol A residues in seafood by high-performance liquid chromatography with tandem mass spectrometry. J. Huazhong Agric. Univ..

[43] Chen Y.L. (2018). SERS Detection Study on Penicillins Drug and Bisphenol A in Milk.

[44] Yang Y., Li D., Liu G., Xu J., Yang L. (2015). Preparation, modification, self-assembly and surface enhanced Raman scattering of gold nanorods and its biomedical application. Sci. Sin. Chim..

[45] Yu X.Y. (2020). Preparation and Modification of Gold Nanostars and Application in SERS Detection.

[46] Ji W., Zhang L., Luo H.S., Song W., Zheng Y., Silver Y. (2016). Nanoparticles Preparation and its SERS study for detection of BPA. J. Light Scat..

[47] Sharma M., Pudasaini P.R., Ruiz-Zepeda F., Vinogradova E., Ayon A.A. (2014). Plasmonic Effects of Au/Ag Bimetallic Multispiked Nanoparticles for Photovoltaic Applications. ACS Appl. Mater. Interfaces.

[48] Li A., Qiao X., Liu K., Bai W., Wang T. (2022). Hollow metal organic framework improves the sensitivity and anti-interference of the detection of exhaled volatile organic compounds. Adv. Funct. Mater..

[49] Huang C., Li A., Chen X., Wang T. (2020). Understanding the role of metal-organic frameworks in surface-enhanced Raman scattering application. Small.

