# Supplementary figures and images for: Application of a Novel Au@ZIF-8 Composite in the Detection of Bisphenol A by Surface-Enhanced Raman Spectroscopy
Source: Foods. 2023 Feb 14;12(4):813. doi: 10.3390/foods12040813 (PMC9956950; doi:10.3390/foods12040813)

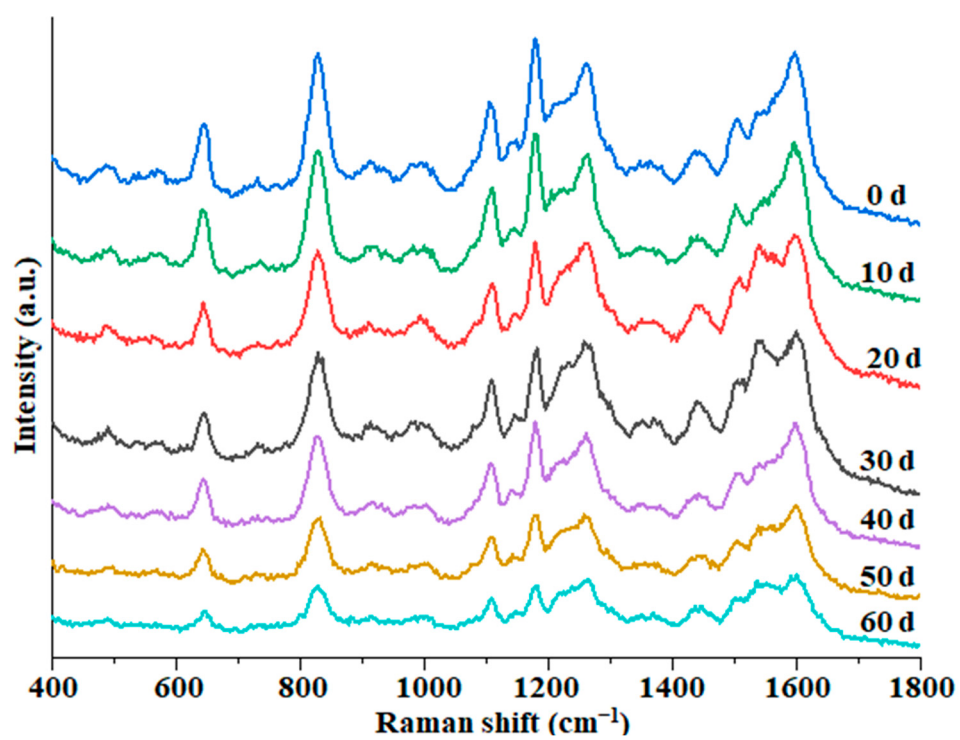

Figure S1. Effect of Au@ZIF-8 on enhancing Raman spectrum signal of BPA with its storage time.

Supplement: Supplementary file 1 [file foods-12-00813-s001.zip › foods-2135523-supplementary.pdf]
